# Flat-IIN: Instance Image Navigation Based on 2D Multi-Channel Semantic Maps and Driven by Vision-Language Models

**DOI:** 10.3390/s26196251

**Published:** 2026-10-01

**Authors:** Weicheng Wang, Rui Lin, Jian Hu, Cong Yu

**Affiliations:** Robotics and Microsystems Research Center, Soochow University, No. 8, Jixue Road, Suzhou 215000, China; 20244229021@stu.suda.edu.cn (W.W.); 20235229100@stu.suda.edu.cn (J.H.); 20255229053@stu.suda.edu.cn (C.Y.)

**Keywords:** instance image navigation, embodied navigation, visual navigation

## Abstract

Instance Image Navigation (IIN), an embodied navigation task, requires an agent to autonomously explore unknown environments and navigate to the vicinity of a target object indicated by an image. The core challenges are efficient semantic exploration without prior maps and accurate target discrimination among similar objects. Existing methods are mainly divided into modular approaches and 3D Gaussian splatting methods; the former lack semantic guidance, while the latter rely on pre-built maps, unsuitable for online navigation. To address this, this paper proposes Flat-IIN, a modular framework using 2D multi-channel semantic maps and a vision-language model (VLM). It constructs multiple 2D maps and integrates Multi-View Active Semantic Exploration, Global Backtracking, and VLM-based Frontier Exploration. These collaboratively leverage the maps to accomplish IIN. Experimental results on the Habitat-Matterport 3D semantic dataset show that Flat-IIN achieves a success rate of 0.794 and success weighted by normalized inverse path length of 0.331. These results demonstrate competitive navigation performance and validate its effectiveness.

## 1. Introduction

In recent years, the rapid advancement of artificial intelligence (AI) has brought about profound transformations in human life and production. Embodied intelligence, as an extension of AI into the physical world, has attracted growing research interest. Embodied intelligence requires an agent to perceive and reason about changes in the external environment in a human-like manner and to interact with the environment to accomplish complex, long-horizon tasks. Embodied navigation serves as a cornerstone for achieving embodied intelligence and can be further decomposed into several subtasks, such as Vision-and-Language Navigation (VLN) [1,2,3,4], Object Goal Navigation (ObjectNav) [5,6,7], and Instance Image Navigation (IIN) [8,9]. Each subtask involves distinct forms of task objectives and instructions, and is suited to different navigation scenarios. This paper focuses on the IIN task, which was defined by Krantz et al. [8] as follows: an agent is randomly initialized in an unknown environment, provided with a reference RGB image containing a specific object instance, and must autonomously explore using its first-person RGB-D observations to finally navigate to the vicinity of the target object instance depicted in the reference image.

The challenges of the IIN task lie in how an agent can efficiently explore an environment without semantic priors and actively navigate to potential target regions, as well as how it can reliably distinguish a target instance from distractors of the same category in complex and dynamic indoor environments—requiring embodied agents to possess strong cross-view object matching and recognition capabilities. Learning-based methods address these challenges through end-to-end training, but suffer from poor generalization and excessive computational cost. Modular methods employ re-identification modules to distinguish target instances from same category distractors, yet they lack effective solutions for efficient exploration and often rely on naive frontier exploration that ignores semantic cues. 3D Gaussian Splatting (3DGS) methods excel at instance identification via novel view rendering, but are inherently dependent on pre-built 3D maps.

**Figure 1:**
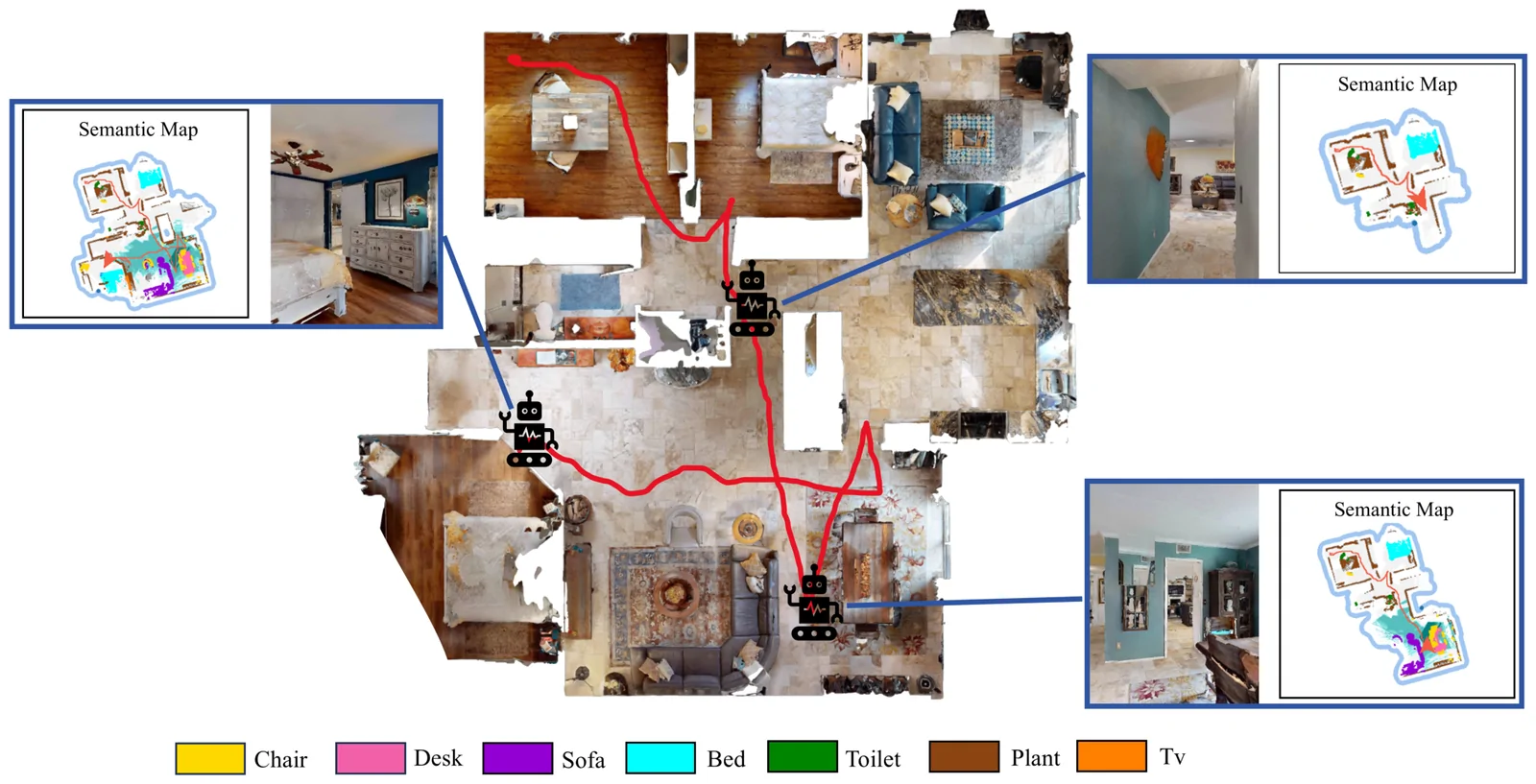
The process of constructing a 2D multi-channel semantic map by the agent. The color coding for object categories is provided in the subscript of the figure. Cyan represents regions with VLM scores; the darker the color, the higher the score. Brown denotes walls, light blue denotes frontier regions, dark blue dots denote target points, and gray denotes obstacles.

To address this highly challenging task, this paper proposes the Flat-IIN framework, which accomplishes the IIN task by constructing 2D semantic maps during navigation and coordinating different mechanisms. The map construction process is illustrated in Figure 1. Accurate matching between the agent’s observations and the target image is critical for instance navigation. However, existing methods often make a decision as soon as an object with similar semantics to the target is detected, lacking the capability to verify from multiple viewpoints. This limitation can easily lead to misjudgment due to restricted viewing angles. To overcome this, Flat-IIN designs a Multi-View Active Semantic Exploration (MASE) mechanism, which enables the agent to actively observe objects of the same semantic class as the target from multiple angles, thereby acquiring more comprehensive visual evidence and more reliably determining whether a candidate object is the true target. Moreover, the target image provided in the IIN task often encodes clear regularities in the spatial distribution of objects. However, existing methods, upon failing to confirm the target after exhaustively exploring the environment, typically only wait passively until the step budget is exhausted, lacking the ability to perform a secondary verification of previously explored areas. To address this, Flat-IIN fully exploits the spatial co-occurrence patterns present in the target image by designing a Global Backtracking (GB) mechanism. This mechanism extracts the categories and quantities of objects in the vicinity of the target as environmental references and, once triggered, leverages the spatial co-occurrence priors between the target object and these references to conduct a secondary verification of candidate targets. Benefiting from the powerful capabilities of VLMs and large language models (LLMs) [10,11,12] in semantic understanding and commonsense reasoning, Flat-IIN introduces a VLM into the IIN task and innovatively assigns it a dual role—both as a semantic guidance signal to drive VLM-based Frontier Exploration (VFE), guiding the agent to efficiently explore unknown environments, and as a semantic discriminator in object detection, collaborating with visual feature similarity to determine the target region, thereby fully leveraging the VLM’s strong capability in cross-view semantic association reasoning.

The specific contributions of this paper are as follows:

1. VLM-based Frontier Exploration mechanism: This mechanism uses VLM semantic reasoning to guide frontier exploration in IIN. The VLM outputs a semantic relevance score between the observation image and the target image. High-score semantic regions are then dilated along navigable areas via morphological dilation, and the exploration value of frontier points is computed by combining geodesic distance weighting. This guides the agent to prioritize frontier regions with high semantic value, significantly improving environment exploration efficiency.
2. Multi-View Active Semantic Exploration mechanism: During navigation, for candidate objects belonging to the same semantic class as the target, a wavefront-expanded observation ring is generated based on the 2D semantic map. A set of discrete multi-view observation points is then obtained via farthest point sampling, allowing the agent to thoroughly observe the candidate object from different viewpoints and thereby more reliably determine whether it is the true target.
3. Global Backtracking mechanism: This mechanism leverages the co-occurrence distribution between the target object and background objects in the target image as a spatial prior. After the agent completes environment exploration, it performs neighborhood semantic verification on instances of the same class in the global semantic map, filtering out candidate regions that satisfy the co-occurrence constraint as targets for secondary verification. This effectively remedies the deficiency of existing methods, which lack backtracking verification capability after exploration is completed.

## 2. Related Work

### 2.1. Instance Image Navigation

In the classic Image Navigation (ImageNav) task, the target image is randomly captured from the environment and often contains regions with little semantic distinctiveness (e.g., walls). Moreover, ImageNav requires the camera parameters used to capture the target image to match those of the navigation agent’s sensors [13,14]. Clearly, the classic ImageNav task is difficult to apply in real-world scenarios. The IIN task addresses these limitations by requiring the target image to focus on a specific object instance and allowing the camera capturing the target image and the agent’s sensor to operate independently of each other. Compared to ImageNav, IIN is better aligned with the demands of real-world settings. Existing IIN methods can be categorized into learning-based approaches [8,15], modular approaches [9,16], and 3DGS methods [17,18]. Learning-based methods primarily rely on deep reinforcement learning (DRL) [19] to train agents that directly map the target image and sensor observations to actions, thereby accomplishing the navigation task. Such approaches typically require enormous computational resources and suffer from limited generalization. For instance, ref. [8] adopts the PPO algorithm [20] framework and trains for 3.5 billion steps using 64 GPUs. Modular methods decompose the IIN task into subtasks such as exploration, target instance re-identification, target localization, and local navigation, and accomplish IIN by addressing each subtask independently. These modules employ off-the-shelf pretrained components without any fine-tuning. Compared to learning-based methods, modular approaches have achieved higher navigation success rates. 3DGS methods utilize 3D Gaussian Splatting [21] to construct a 3D scene representation rich in geometry, semantics, and texture, and then employ differentiable rendering to generate images of the target object from arbitrary viewpoints. By matching these rendered images with the given single target image, the target instance can be directly localized. Although 3DGS methods can provide textural details, they incur high computational cost and heavily rely on continuous, high-quality online mapping.

### 2.2. Visual Navigation Based on Large Foundation Models

In recent years, VLMs and LLMs have advanced rapidly. Owing to their powerful capabilities in semantic understanding, commonsense reasoning, and zero-shot generalization, they offer new paradigms for tackling challenges in visual navigation, such as environment perception, instruction comprehension, and action planning. For instance, refs. [22,23,24,25] adopt LLMs as high-level planners, leveraging their rich commonsense priors and logical reasoning abilities to perform instruction decomposition and spatial relation reasoning. By converting complex visual and geometric information into structured prompts that are easily interpretable by large models, these methods decouple high-level semantic cognition from low-level motion control, endowing agents with the ability to "think before acting" in unknown environments. In addition, ref. [26] focuses on VLN and proposes NavCoT, which leverages parameter-efficient in-domain training to enable an LLM to perform navigation reasoning through a three-stage decoupled chain of thought. Ref. [27] introduces DiscussNav, a zero-shot navigation framework that no longer relies on a single model for independent decision-making. Instead, multiple LLMs are assigned as experts in different domains via role prompts, and before each action, the navigation agent actively engages in multiple rounds of discussion with these experts to decompose navigation subtasks. Unlike LLMs that primarily focus on text-based reasoning, VLMs inject visual perception capabilities natively into language models, allowing them to directly process environmental images or video streams [28,29,30,31]. For example, ref. [29] focuses on ObjectNav and combines a VLM with a world model to improve navigation efficiency and mitigate VLM hallucinations by predicting future environment states. Ref. [31] adopts a dual-VLM hierarchical approach, where a Reasoning-VLM performs instruction semantic parsing and global scene reasoning to identify the target area, and a specifically fine-tuned Pointing-VLM handles local perception and precise localization of complex spatial relations, which is then combined with 3D scene representations to accomplish long-range hierarchical navigation.

## 3. Instance Image Navigation Preliminaries

Observation Space: At each timestep *t*, the agent’s observation $O_{t}=\left\{I^{*},I_{t},D_{t},P_{t}\right\}$. $I^{*}$ is the target RGB image, $I_{t}$ and $D_{t}$ are respectively the agent’s egocentric RGB and depth images at timestep *t*, and $P_{t}=\left(x,y,\theta \right)$ denotes the agent’s pose relative to the initial pose $P_{0}=\left(0,0,0\right)$ at timestep *t*. Following the setup of Krantz et al. [8], the camera parameters for capturing the target image $I^{*}$ and the agent’s egocentric camera are independent. The target image $I^{*}$ has a resolution of 512×512, while the agent’s egocentric RGB image $I_{t}$ and depth image $D_{t}$ have a resolution of 640×480 with a horizontal field of view (FOV) of $58^{\circ }$.

Action Space: This paper adopts a discrete action space. At each timestep *t*, the agent’s action $A_{t}=\left\{\mathrm{MOVE}\text{-}\mathrm{FORWARD},\mathrm{TURN}\text{-}\mathrm{LEFT},\mathrm{TURN}\text{-}\mathrm{RIGHT},\mathrm{STOP}\right\}$. The step size for moving forward is 0.25 m, and the rotation angle for a single turn is $30^{\circ }$.

## 4. Methodology

**Figure 2:**
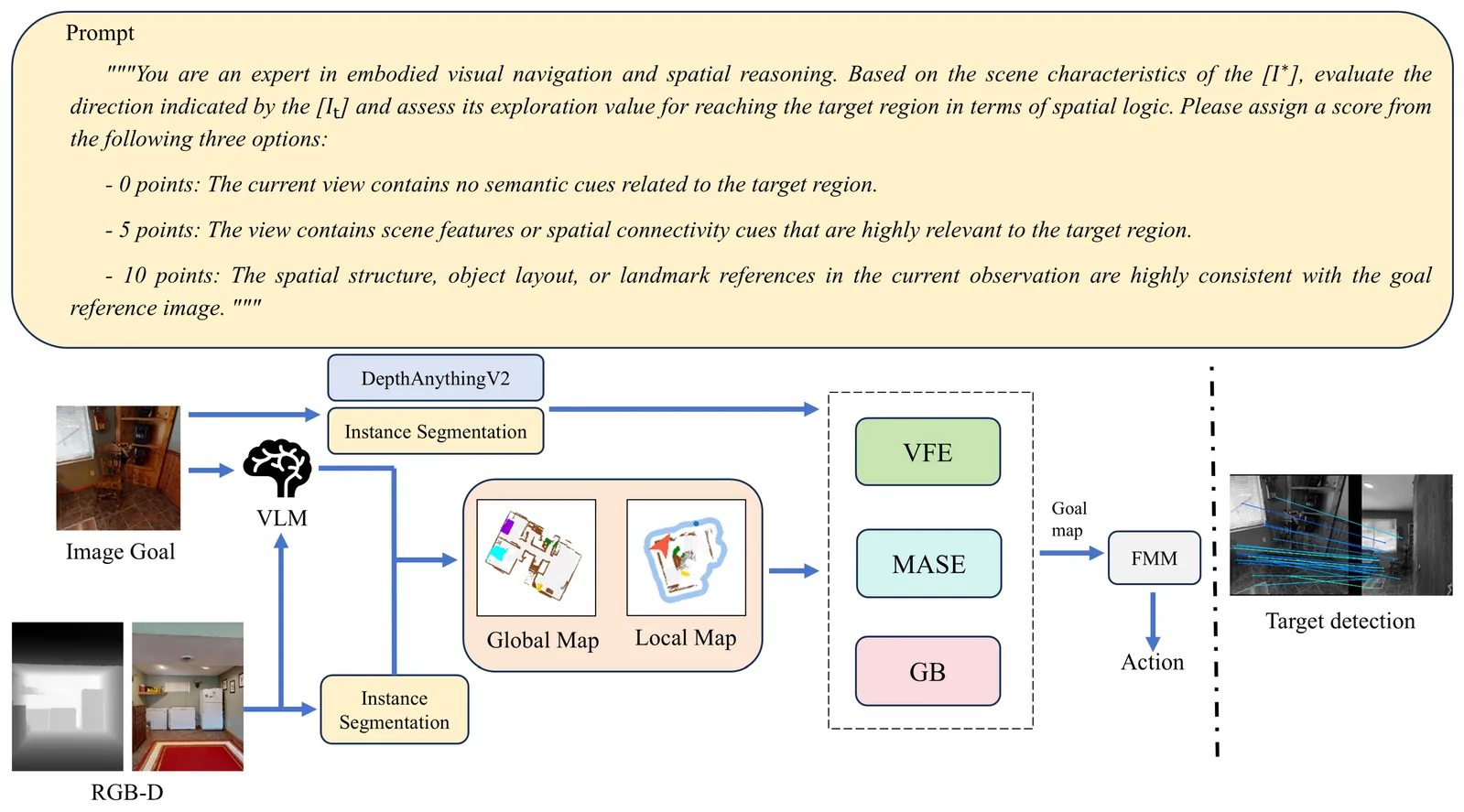
Overall pipeline of Flat-IIN.

The overall pipeline of Flat-IIN is illustrated in Figure 2. Based on its egocentric RGB-D observations, Flat-IIN first passes the data through a VLM and an instance segmentation model to construct local and global semantic maps. The agent then employs the VFE mechanism to explore the environment efficiently. Upon detecting objects of the same semantic class as the target, the MASE mechanism is triggered to observe the candidate object from multiple viewpoints and determine whether it is the true target. If the entire environment has been explored without successfully identifying the target object, the GB mechanism is invoked to re-observe previously explored candidate objects of the same class. After a target map is obtained through VFE, MASE, and GB, the Fast Marching Method (FMM) [32] is applied to generate actions. During the navigation process, dual-condition object detection is performed on each observation image against the target image, computing a semantic relevance score between them. If the score exceeds a predefined threshold, the current observation is labeled as a target region.

### 4.1. Construction of Multi-Channel Semantic Maps

Based on the mapping method proposed in [5], Flat-IIN constructs an egocentric multi-channel 2D local semantic map $M_{\mathrm{local}}^{t}\in R^{C\times W\times H}$, where *W* and *H* denote the width and height of the semantic map, both set to 480, with a map resolution of 5cm per cell. C=8+N is the number of channels, where *N* represents the number of object categories from instance segmentation. At each timestep *t*, the agent receives egocentric RGB-D observations. For the RGB image, a pretrained instance segmentation model is applied to obtain the segmentation result $I_{\mathrm{seg}}^{t}$. Meanwhile, using the camera intrinsic matrix *K* and the current camera pose, the depth map $D_{t}$ is back-projected into a 3D point cloud $Q^{t}$, which is then discretized into a 3D voxel grid $V^{t}$. Feature projection from 3D to 2D is achieved by aggregating features along the height axis (*Z*-axis). For the map channels obtained via voxel projection, the feature value of the 2D local map $M_{\mathrm{local}}^{t}$ at coordinate (x,y) is computed by the following formula:

(1) $$M_{\mathrm{local}}^{t}\left(x,y,c_{i}\right)=\sum_{z\in H_{c_{i}}}V^{t}\left(x,y,z,c_{i}\right)$$

where $H_{c_{i}}$ denotes the height slice interval partitioned for the *i*-th channel, and $c_{i}$ represents the *i*-th channel.

Based on this projection paradigm and spatial logic, the multi-channel local semantic map constructed in this paper includes not only the obstacle map $M_{\mathrm{obs}}$, the explored area map $M_{\exp}$, the current position map $M_{\mathrm{loc}}$, and the historical trajectory map $M_{\mathrm{vis}}$, but also the following maps:

Wall map $M_{\mathrm{wall}}$: The point cloud $Q^{t}$ is processed using iterative RANSAC to extract multiple vertical planes. The inlier points of the detected planes are regarded as wall points and projected onto the 2D map to construct $M_{\mathrm{wall}}$.

Object category map $M_{\mathrm{sem}}$: The points in the point cloud $Q^{t}$ are associated with the segmentation result $I_{\mathrm{seg}}^{t}$, thereby endowing the point cloud with instance-level semantic features, which are subsequently projected onto the 2D plane along with the voxels to form $M_{\mathrm{sem}}$.

Goal map $M_{\mathrm{goal}}$: The keypoints matched between the current observation image and the target image are associated with the point cloud $Q^{t}$ and then mapped onto the 2D map as $M_{\mathrm{goal}}$.

VLM score map $M_{\mathrm{vlm}}$: The scalar score $S_{\mathrm{vlm}}$ output by the VLM is used to update the corresponding positions of $M_{\mathrm{vlm}}$ based on the physical visibility mask of the current frame (i.e., the grid cells covered by the projection of the camera frustum onto the 2D map at timestep *t*), by taking the element-wise maximum across observations.

Blacklist map $M_{\mathrm{black}}$: To prevent the agent from becoming stuck, Flat-IIN designates the region around the current target point on the local map as a blacklist area if the agent remains stationary for more than a predefined number of consecutive steps; this area is then excluded from subsequent target point selection.

### 4.2. Object Detection with Dual-Condition Mechanism

Flat-IIN employs a dual-condition mechanism to determine whether the agent has arrived near the target region. One of the criteria follows the approach in [9]: using the pretrained SuperPoint [33] model, keypoints and their descriptors are extracted from both the target image $I^{*}$ and the observation image $I_{t}$. These are then fed into the SuperGlue [34] model for feature matching, and a local feature matching score $S_{\mathrm{glue}}$ is obtained through aggregation:

(2) $$S_{\mathrm{glue}}=\mathrm{SuperGlue}\left(\mathrm{SuperPoint}\left(I^{*}\right),\mathrm{SuperPoint}\left(I_{t}\right)\right)$$

For the second criterion, the score $S_{\mathrm{vlm}}$ is first retrieved from the region in the VLM score map $M_{\mathrm{vlm}}$ corresponding to the goal map $M_{\mathrm{goal}}$. Meanwhile, the DINOv2 [35] visual model is employed to extract the target image features $F^{*}$ and the observation image features $F^{t}$, and the cosine similarity score $S_{\mathrm{dino}}$ between $F^{*}$ and $F^{t}$ is computed:

(3) $$S_{\mathrm{dino}}=\frac{F^{*}\cdot F^{t}}{∥F^{*}∥\;∥F^{t}∥}$$

The final semantic score $S_{\mathrm{sem}}$ is obtained by computing a weighted sum of the raw scores $S_{\mathrm{glue}}$, $S_{\mathrm{vlm}}$, and $S_{\mathrm{dino}}$:

(4) $$S_{\mathrm{sem}}=\alpha S_{\mathrm{glue}}+\beta S_{\mathrm{vlm}}+\gamma S_{\mathrm{dino}}$$

where α, β, and γ are weighting coefficients.

The final decision logic is as follows. When $S_{\mathrm{glue}}$ alone exceeds the predefined threshold, the target is directly confirmed without relying on semantic fusion. When local feature matching is insufficient for reliable identification, $S_{\mathrm{sem}}$ serves as a complementary criterion. Specifically, if either $S_{\mathrm{glue}}$ or $S_{\mathrm{sem}}$ exceeds its predefined threshold, the agent is considered to have found the target image, and $M_{\mathrm{goal}}$ is assigned as the final target region:

(5) $$\mathrm{FoundGoal}=\left\{\begin{array}{cc} \mathrm{True}, & \left(S_{\mathrm{glue}}>\tau_{1}\right)\vee \left(S_{\mathrm{sem}}>\tau_{2}\right),\\ \mathrm{False}, & \mathrm{otherwise}. \end{array}\right.$$

where $\tau_{1}$ and $\tau_{2}$ are preset score thresholds, respectively.

### 4.3. VLM-Driven Frontier Exploration

Experiments indicate that the VLM can infer from a considerable distance whether the area ahead is likely to correspond to the region depicted in the target image $I^{*}$. Accordingly, Flat-IIN feeds the observation image $I_{t}$ and the target image $I^{*}$ into the VLM and queries the exploration value of the current heading direction using the prompt illustrated in Figure 2. The VLM outputs a discrete semantic score $S_{\mathrm{vlm}}\in \left\{0,5,10\right\}$, where the values represent “no semantic cues,” “semantically relevant,” and “strong semantic alignment,” respectively. The obtained score $S_{\mathrm{vlm}}$ is then used to update the VLM score map $M_{\mathrm{vlm}}$ based on the physical visibility mask of the current frame.

Flat-IIN first computes the boundary between explored and unexplored areas based on the explored map $M_{\exp}$, and designates it as the initial frontier region *F*. The classical frontier-based exploration strategy [36] simply selects the nearest frontier as the target point, ignoring the semantic information of frontier regions. Therefore, Flat-IIN applies morphological dilation to $M_{\mathrm{vlm}}$ to propagate the high-score regions in $M_{\mathrm{vlm}}$ outward. To prevent misleading guidance through walls, the dilation process is strictly constrained by the wall map $M_{\mathrm{wall}}$, i.e., $M_{\mathrm{vlm}}$ is not allowed to cross walls. The dilation is performed for at most 40 iterations, and the propagation state at the *k*-th iteration is expressed as:

(6) $$M_{\mathrm{vlm}}^{k}=\mathrm{Dilate}\left(M_{\mathrm{vlm}}^{k-1},K_{\mathrm{morph}}\right)⊙\left(1-M_{\mathrm{wall}}\right)$$

where $K_{\mathrm{morph}}$ is a 3×3 morphological dilation kernel.

By intersecting the dilated $M_{\mathrm{vlm}}$ with the original frontier *F*, each original frontier point f∈F is assigned a VLM score, denoted as $S_{\mathrm{vlm}}\left(f\right)$. Next, the geodesic distance d(f) from the agent’s current position to each frontier point *f* is computed on the obstacle map $M_{\mathrm{obs}}$. The exploration utility U(f) of each frontier candidate point *f* is then obtained through a weighted sum of $S_{\mathrm{vlm}}\left(f\right)$ and d(f):

(7) $$U\left(f\right)=w_{\mathrm{sem}}\cdot S_{\mathrm{vlm}}\left(f\right)-w_{\mathrm{dist}}\cdot d\left(f\right)$$

where $w_{\mathrm{sem}}$ and $w_{\mathrm{dist}}$ are the weights for semantic exploration and path cost, respectively.

Flat-IIN then selects the frontier point $f^{*}$ with the maximum exploration utility as the navigation target at the current moment:

(8) $$f^{*}=\arg\max_{f\in F}U\left(f\right)$$

### 4.4. Multi-View Active Semantic Exploration

For the target image $I^{*}$, Flat-IIN employs Detic [37], an open-vocabulary object detector, to perform instance segmentation and selects the object instance located at the center of the image with the highest confidence as the target object $T_{\mathrm{obj}}$. The local semantic map corresponding to the object category of $T_{\mathrm{obj}}$ is denoted as $M_{\mathrm{local}}^{\mathrm{obj}}$, where $M_{\mathrm{local}}^{\mathrm{obj}}\subset M_{\mathrm{sem}}$. To prevent ineffective target identification due to limited viewpoints, Flat-IIN performs iterative wavefront expansion on $M_{\mathrm{local}}^{\mathrm{obj}}$ for 8 iterations, thereby generating an observation ring $R_{\mathrm{obs}}$ at a safe radius of approximately 40cm from the object. Farthest point sampling is then applied to the traversable regions on $R_{\mathrm{obs}}$, with a minimum distance of 50cm between sampled viewpoints, yielding at most 5 discrete observation points $P_{\mathrm{sample}}$. Let the initial wave source be $w^{0}=M_{\mathrm{local}}^{\mathrm{obj}}$; after *k* iterations of expansion, the observation ring is expressed as:

(9) $$R_{\mathrm{obs}}=\left(w^{k}-w^{k-1}\right)⊙M_{\mathrm{allowed}}$$

where $w^{k}$ is the wavefront set after the *k*-th dilation, and $M_{\mathrm{allowed}}$ is the medium mask that permits the wavefront to reside. After binarizing the required semantic maps involved in the computation, $M_{\mathrm{allowed}}$ is defined as follows:

(10) $$M_{\mathrm{allowed}}=\left(M_{\exp}\vee M_{\mathrm{obs}}\vee M_{\mathrm{sem}}\right)\wedge \left(1-M_{\mathrm{vis}}\right)\wedge M_{\mathrm{vlm}}$$

**Figure 3:**
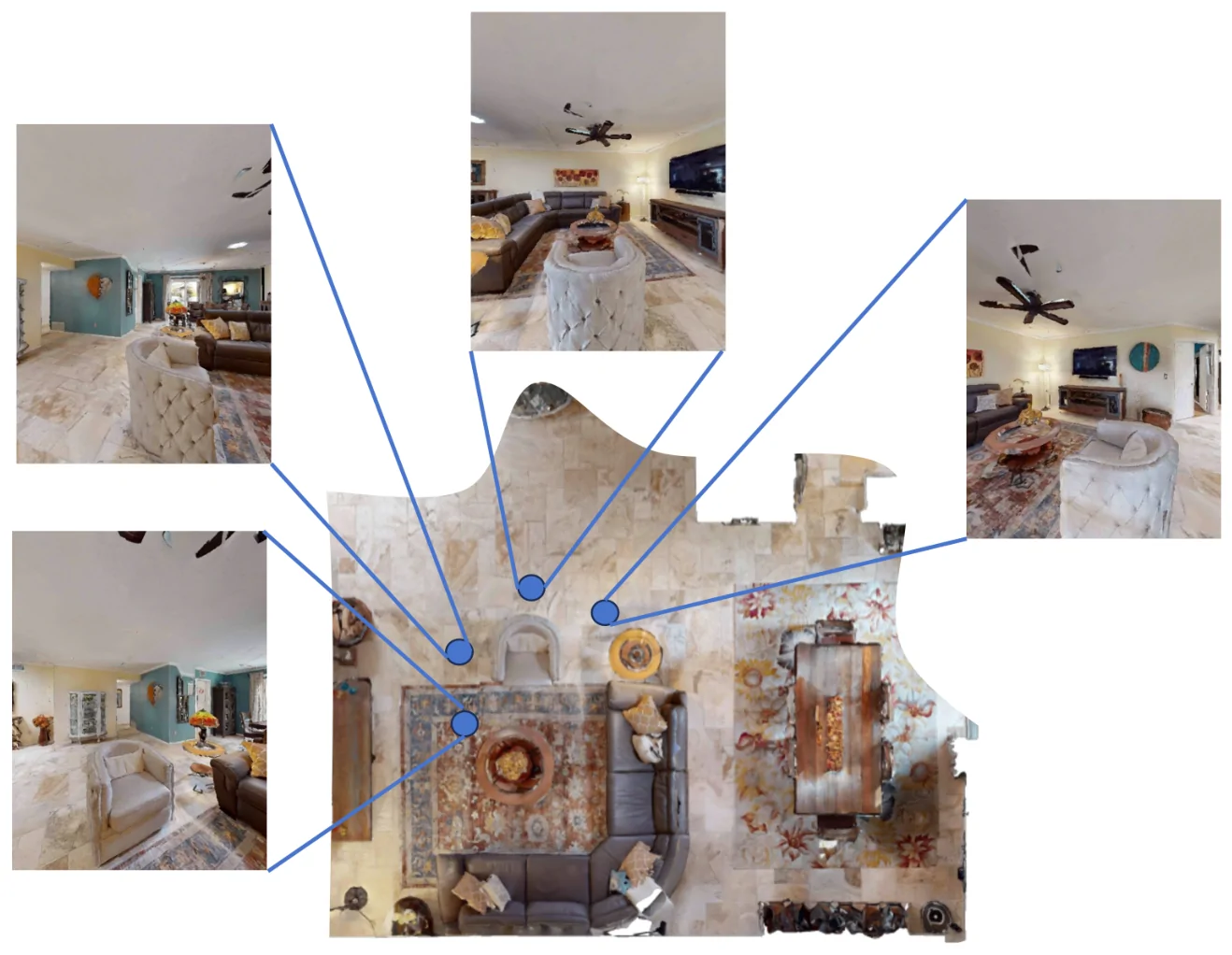
Illustration of multi-view active semantic exploration.

In Equation (10), the obstacle map $M_{\mathrm{obs}}$ is incorporated into the allowed-region mask $M_{\mathrm{allowed}}$ via a logical OR operation, so that the wavefront can temporarily reside on obstacle cells during MASE expansion. This does not mean that observation points are placed on obstacles. Non-traversable cells are removed at the final observation-point sampling stage, where farthest point sampling is performed only over traversable regions of the generated observation ring. Therefore, obstacles can serve as a propagation medium, but are never selected as observation viewpoints. During navigation, as new $P_{\mathrm{sample}}$ points are obtained, they are set as the current navigation targets, thereby enabling multi-view observation of the target object. Figure 3 illustrates the process of multi-view object observation via observation point generation.

### 4.5. Global Backtracking

To achieve GB, Flat-IIN simultaneously maintains a multi-channel global semantic map $M_{\mathrm{global}}$. Both its length and width are 960, its resolution is 5cm per grid cell, and its construction logic is consistent with that of the local map $M_{\mathrm{local}}$. Based on $M_{\mathrm{local}}$, the GB mechanism is triggered when the agent determines that there are no physically reachable exploration frontiers on the map.

Flat-IIN employs DepthAnythingV2 [38], a monocular depth estimation model, to extract the median depth of the target object $T_{\mathrm{obj}}$ in the target image $I^{*}$. It then identifies the background object categories and their counts in $I^{*}$ that lie at the same depth level as $T_{\mathrm{obj}}$, i.e., objects whose normalized depth value differs from that of the target object by less than 60, denoted as $\Omega \left(I^{*}\right)=\left\{\left(o_{k},n_{k}\right)\right\}$, where $o_{k}$ represents the *k*-th object category and $n_{k}$ denotes the number of instances of that category in $I^{*}$. When the GB mechanism is triggered, the semantic map $M_{\mathrm{global}}^{\mathrm{obj}}$ corresponding to the object category of $T_{\mathrm{obj}}$ is extracted from $M_{\mathrm{global}}$. Global candidate instances are obtained through morphological denoising and connected component analysis. For each global candidate instance, a neighborhood window $N(r_{i},R)$ of side length 2R is defined, where $r_{i}$ is the centroid of the *i*-th object. The semantic feature distribution of various categories within this neighborhood window is denoted as $\Omega \left(N_{i}\right)=\left\{\left(o_{k},{\hat{n}}_{k}\right)\right\}$, with ${\hat{n}}_{k}$ being the count of object category $o_{k}$. An instance $e_{i}$ is designated as a backtracking target if and only if $\Omega (N_{i})$ satisfies the co-occurrence constraint $\Omega (I^{*})$. This is formulated as follows:

(11) $$e_{i}\in \mathrm{Target}\Leftrightarrow \forall \left(o_{k},n_{k}\right)\in \Omega \left(I^{*}\right),\;{\hat{n}}_{k}\geq n_{k}$$

**Figure 4:**
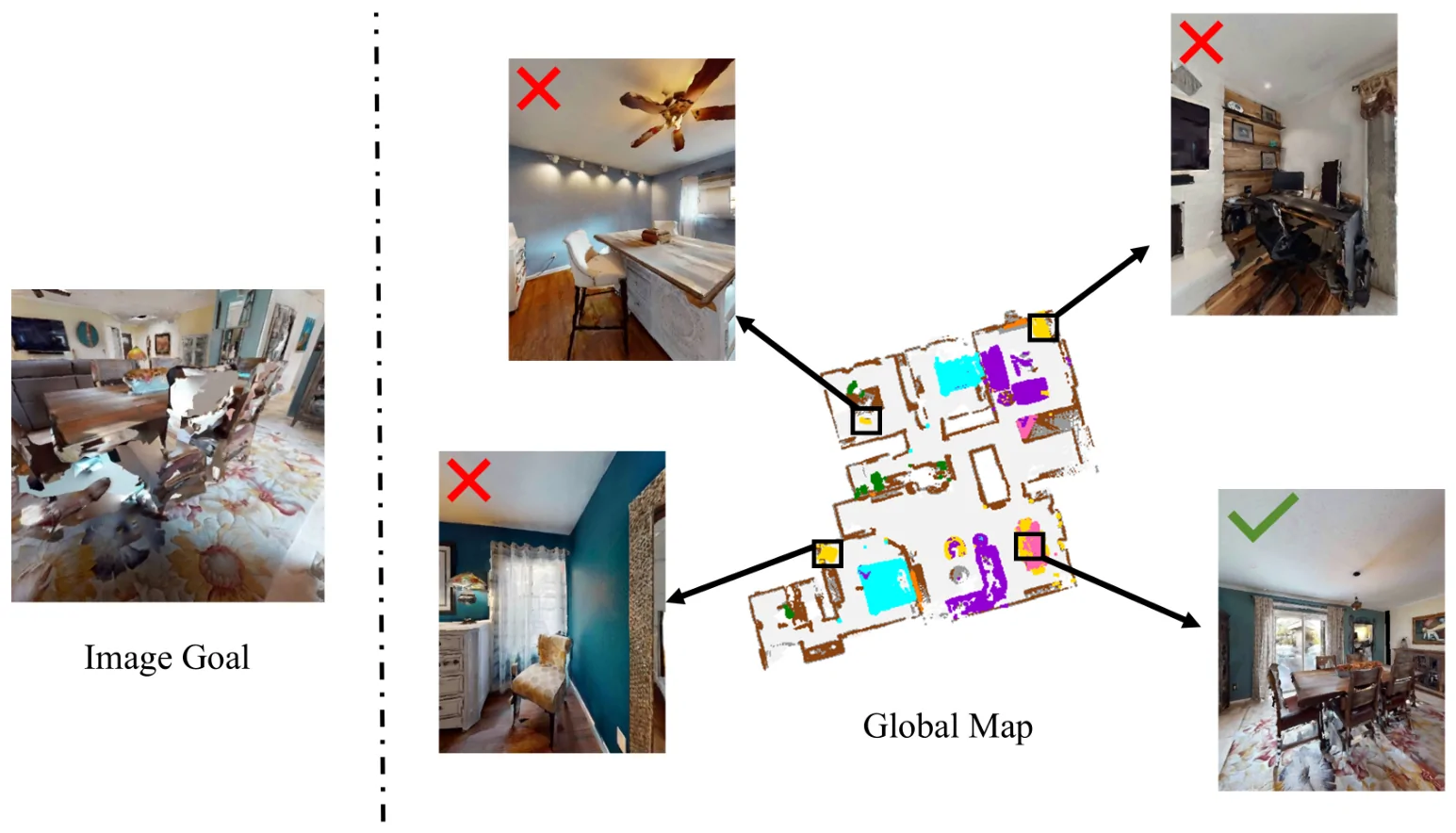
Illustration of the GB mechanism. After applying Detic instance segmentation to the target image, the target object is identified as a chair. After depth filtering of the target image using DepthAnythingV2, the background objects are determined to include three chairs and one table. When GB is triggered, the global candidate instances are screened, and those satisfying the co-occurrence constraint are selected as backtracking targets. Cross: constraint not satisfied; check: satisfied.

Finally, after all backtracking instances have been revisited, the $M_{\mathrm{goal}}$ region with the highest semantic score $S_{\mathrm{sem}}$ is designated as the final target region. Figure 4 illustrates the GB mechanism.

### 4.6. Local Navigation

Flat-IIN adopts the FMM as its local navigation approach, which has been widely used in embodied navigation tasks. Once a navigation target point is obtained, the FMM algorithm takes this target point as the wave source and simulates the wavefront propagation process over the obstacle map, computing the arrival time field from the target point to all grid cells across the map. Then, starting from the agent’s current position, the path is traced back along the gradient descent direction of the time field, yielding a shortest-time path that avoids obstacles. Based on this path, the agent selects actions from the action space $A_{t}$ to progressively approach the target.

## 5. Experiments

All experiments in this paper were conducted on Ubuntu 20.04, with an NVIDIA RTX 4060 GPU and an Intel Core i7-14650HX CPU. The relevant parameters involved in the experiments were tuned on the training set of the episode dataset for the IIN task, as shown in Table 1.

### 5.1. Experimental Setup

Dataset: The simulation experiments in this paper are conducted on the Habitat [39] simulation platform. The experimental scenes use the semantically annotated Habitat-Matterport 3D dataset [40,41], which contains 216 scenes divided into training/validation/test sets with 145/36/35 scenes, respectively. The episode dataset for the IIN task is generated according to the IIN rules proposed by Krantz et al. [8], requiring that all episodes are restricted to single-floor navigation. The IIN task episodes are split into training, validation, and test sets, with sizes of 7056 K, 1 K, and 1 K episodes, respectively. The target images cover six object categories: {chair,sofa,plant,bed,toilet,TV}.

Embodied Agent: In the Habitat simulation environment, the agent is configured as a rigid-body cylinder with zero turning radius, a height of 1.41 m, and a radius of 0.17 m. A front-facing RGB-D camera is mounted at a height of 1.31 m. For the real-world deployment scenario, we use a Turtlebot2 as the physical verification platform, which has a radius of 0.17 m and is equipped with an Orbbec Gemini 336L camera mounted at a height of 0.88 m.

Evaluation Metrics: In this paper, Flat-IIN is primarily evaluated from two aspects: Success Rate (SR) and Success rate weighted by normalized inverse Path Length (SPL). An episode is judged as successful (Success = 1) if the Euclidean distance between the agent and the target object is less than 1.0 m when the STOP action is issued. SPL is an efficiency metric for navigation tasks, defined by [42] as:

(12) $$\mathrm{SPL}=\frac{1}{N}\sum_{i=1}^{N}S_{i}\frac{l_{i}}{\max(p_{i},l_{i})}$$

where *N* is the total number of episodes, $S_{i}\in \left\{0,1\right\}$ indicates whether the *i*-th episode is successful, $l_{i}$ is the shortest path distance from the start to the target, and $p_{i}$ is the actual path length traveled by the agent. A higher SPL indicates greater navigation efficiency. The maximum step limit is set to 1000 steps. If the agent’s navigation steps exceed 1000, the episode is judged as a timeout failure.

### 5.2. Ablation Study on Different Model Combinations

To investigate the impact of different instance segmentation models and VLMs on the SR and SPL of Flat-IIN, this paper selects Mask R-CNN [43] and the combination of YOLOv7 [44] + MobileSAM [45] as the instance segmentation models, and GLM [46] and Qwen [47] as the VLMs. GLM refers to GLM-4.6V, and Qwen refers to Qwen-VL-Plus. For each test, 100 episodes are independently and randomly sampled from the training set, and the experiment is repeated for 5 rounds. In each round, all model combinations are evaluated on the same 100 episodes. Finally, for each combination, the average SR and SPL over the 5 rounds are computed and reported in Table 2. As can be seen from Table 2, among all model combinations, the combination of YOLOv7 + MobileSAM and GLM achieves the best performance in terms of SR (0.806) and SPL (0.346). Therefore, Flat-IIN adopts YOLOv7 + MobileSAM as the instance segmentation model and GLM-4.6V as the VLM.

Meanwhile, to determine the optimal VLM invocation frequency, this paper designs a comparative experiment to evaluate the SR, SPL, and other metrics of Flat-IIN under different invocation frequencies. The experiment consists of 5 rounds. In each round, 100 different episodes are independently and randomly sampled from the training set. Within each round, all invocation frequencies are evaluated on the same 100 episodes. Finally, for each invocation frequency, the average value over the 5 rounds is computed, and the results are reported in Table 3. Here, FPS denotes the frame rate during navigation, Token denotes the VLM token consumption per episode, and Frame Interval denotes the number of frames between two consecutive VLM calls. As can be seen from Table 3, as the Frame Interval increases (i.e., the VLM invocation frequency decreases), FPS gradually increases and token consumption drops significantly, indicating that reducing the invocation frequency can effectively improve navigation real-time performance and reduce computational cost. However, SR and SPL do not vary monotonically with frequency; the optimal values are achieved when the Frame Interval is 3 (SR = 0.798, SPL = 0.351). Therefore, Flat-IIN sets the VLM invocation Frame Interval to 3.

### 5.3. VLM Semantic Score Perturbation Sensitivity Analysis

To evaluate the sensitivity of Flat-IIN to the VLM output semantic score, we conduct a perturbation experiment on the discrete VLM score. Specifically, with probability *p*, the current VLM score is replaced by one of the other two possible values chosen uniformly at random; with probability 1−p, it remains unchanged. We test p∈{0.0,0.1,0.2,0.3,0.4,0.5}, where p=0 denotes the unperturbed baseline. The experiment consists of 5 rounds. In each round, 100 different episodes are independently and randomly sampled from the validation set to evaluate different perturbation probabilities. Finally, for each perturbation probability, the average SR and SPL over the 5 rounds are computed, and the results are shown in Table 4.

Table 4 shows that as the perturbation probability *p* increases from 0 to 0.5, SR decreases from 0.776 to 0.746, and SPL decreases from 0.322 to 0.282. This indicates that VLM score perturbations have a more pronounced impact on exploration efficiency. Erroneous VLM scores can mislead the agent to select suboptimal frontier points, resulting in an increase in ineffective exploration paths.

### 5.4. Effectiveness of Dual-Condition Object Detection

To verify that the dual-condition object detection can effectively reduce false negatives during the exploration process (i.e., cases where the agent has reached the vicinity of the target region but fails to recognize it), we design a comparative experiment with two settings: dual-condition and single-condition object detection. The single-condition setting follows the approach proposed in [9] and makes decisions based solely on SuperGlue feature matching. The dual-condition setting incorporates, on top of SuperGlue, the VLM semantic score and DINOv2 visual similarity. Both settings retain the GB mechanism and select the $M_{\mathrm{goal}}$ region with the highest semantic score $S_{\mathrm{sem}}$ as the final target region. For each test, 100 episodes are independently and randomly sampled from the validation set, and the experiment is repeated for 5 rounds. In each round, all conditions are evaluated on the same 100 episodes. Finally, for each condition, the average SR and average SPL over the 5 rounds are computed and reported in Table 5.

As shown in Table 5, the dual-condition setting achieves a higher SPL than the single-condition setting. This result indicates that the single-condition setting is more prone to false-negative detections when initially approaching the target, and thus must rely on the secondary backtracking provided by the GB mechanism to finally confirm the target, thereby increasing the length of ineffective exploration paths.

### 5.5. Comparison with State-of-the-Art Methods

In this section, we categorize the main existing IIN methods into three types: end-to-end methods, modular methods, and 3DGS methods. We compare Flat-IIN with these state-of-the-art approaches in terms of SR and SPL. Table 6 shows the comparison results on the validation set over 1000 episodes. The results for all compared methods are cited from their respective original papers.

- End-to-end methods: End-to-end methods primarily rely on DRL training to directly map observations to actions. RL Baseline [8] encodes RGB-D observations and the target image with a ResNet50, concatenates them with GPS and heading information, and feeds the result into a two-layer LSTM network. It is trained using PPO and directly outputs navigation actions. OVRL-v2-Image [15] similarly takes RGB observations and the target image as input, processes them through an LSTM, and is trained with DD-PPO. OVRL-v2-IIN is obtained by fine-tuning OVRL-v2-Image on the IIN task.
- Modular methods: Modular approaches decompose the IIN task into several subtasks. Mod-IIN [9] first proposed a modular method for IIN, dividing the task into four modules—exploration, re-identification, localization, and local navigation—and employing off-the-shelf components without any training. IEVE [16] decouples IIN into a three-stage “exploration–verification–exploitation” pipeline, where a switching policy dynamically changes the behavioral mode based on the number of matched keypoints and distance, enabling the agent to approach and confirm the target instance. UniGoal [48] is a general zero-shot goal navigation framework that introduces an LLM to unify scenes and goals into graph-structured representations, measures overlap through graph matching, and dynamically switches among three exploration strategies based on the matching status.
- 3D Gaussian Splatting methods: These methods primarily rely on 3DGS to pre-build a scene representation, thereby accomplishing the IIN task. GaussNav [17] first introduced 3DGS into visual navigation, replacing 2D bird’s-eye-view (BEV) maps with a semantic Gaussian map while preserving the scene’s 3D geometric information, semantic labels, and object texture details. It renders multi-view images of candidate instances belonging to the same category and matches them with the target image. GauScoreMap [49] proposes a two-level scoring mechanism: global semantic scoring filters candidate regions, and local geometric scoring precisely localizes the target instance. This approach avoids exhaustive or random sampling of multi-view rendered images, significantly reducing computational cost while improving localization accuracy.

As shown in Table 6, Flat-IIN achieves an SR of 0.794, comparable to that of the best 3DGS method GauScoreMap (0.784), while higher than that of the best modular method IEVE (0.684). Although the SR is comparable to that of GauScoreMap, our main advantage is that Flat-IIN does not require a pre-built global map. The SPL of Flat-IIN (0.331) is lower than that of the 3DGS methods (0.578 and 0.605), because 3DGS methods reuse a scene-level map: a single pre-built Gaussian map is shared across multiple episodes in the same scene, avoiding exploration from scratch for each episode and allowing subsequent episodes to directly use the existing map for target localization and path planning. In contrast, Flat-IIN follows the IIN task definition and does not rely on a pre-built global map; each episode requires online perception and planning. Nevertheless, Flat-IIN achieves an SR comparable to the best 3DGS method and the best SPL among the evaluated methods that do not rely on pre-built scene representations.

To enable a fair comparison under the same setting, this paper takes GaussNav as an example and conducts a comparative experiment. 100 episodes are randomly selected, and Flat-IIN is compared with an online version of GaussNav. Specifically, GaussNav is required to first construct a complete 3DGS map of the environment in each episode, and then perform target search and navigation, with its mapping cost included in the path length. The navigation results are as follows: Flat-IIN achieves an SR of 0.76 and an SPL of 0.356, while online GaussNav achieves an SR of 0.55 and an SPL of 0.085. This result indicates that once cross-episode map reuse is removed, GaussNav’s SPL advantage disappears.

### 5.6. Ablation Study on Navigation Mechanisms

**Figure 5:**
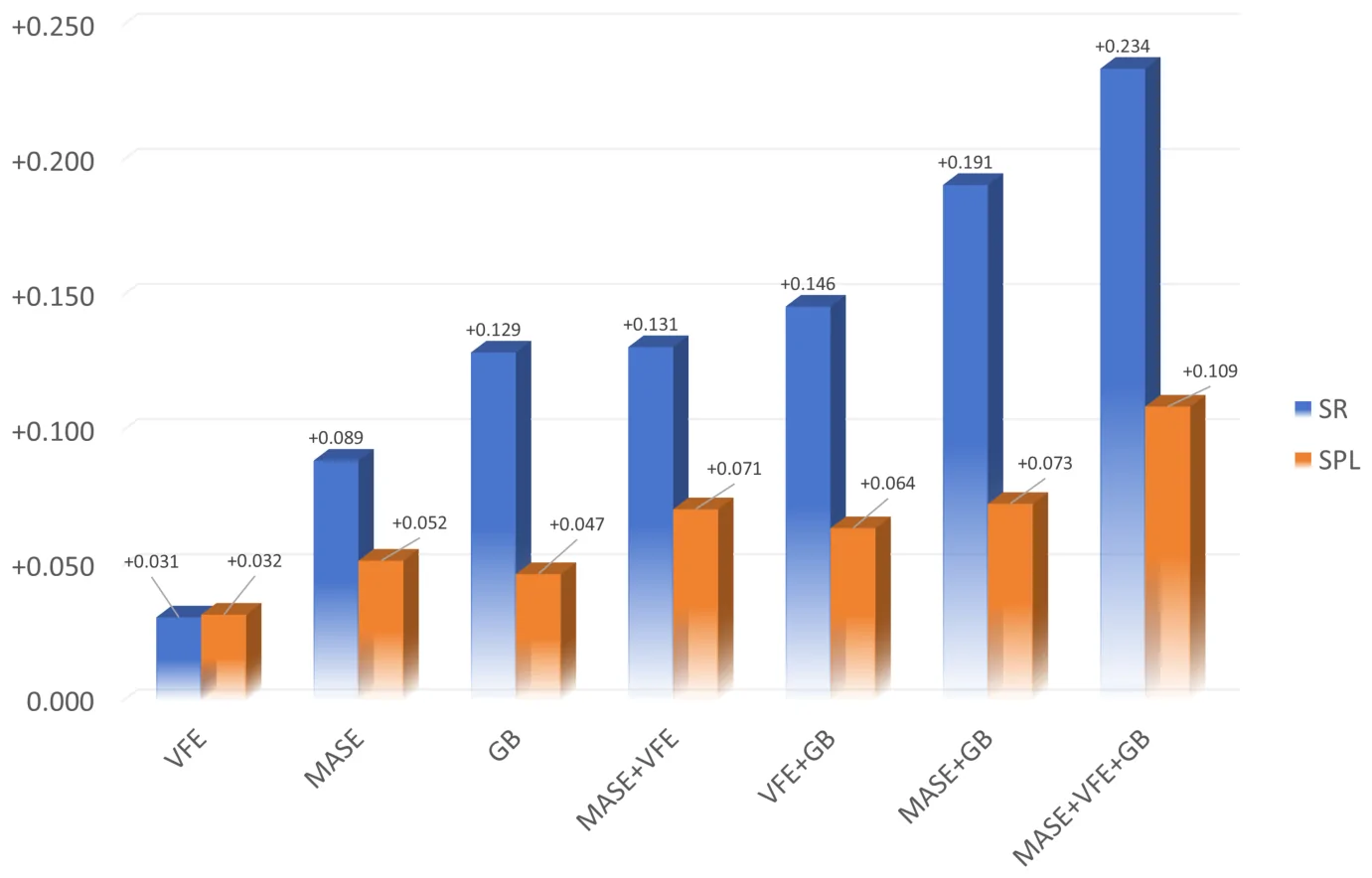
Comparison of the performance improvements contributed by each mechanism to Flat-IIN.

To evaluate the impact of MASE, GB, and VFE on the navigation performance of Flat-IIN, we conduct an ablation study. By testing different combinations of these mechanisms, we verify their individual effects on the SR and SPL of Flat-IIN. After evaluating 1000 episodes on the validation set, the detailed data are presented in Table 7. Moreover, to quantify the contribution of each mechanism to the overall performance, we take the model without any of these mechanisms as the baseline (SR = 0.560, SPL = 0.222) and compute the performance improvement contributed by each mechanism individually, as illustrated in Figure 5.

From Table 7 and Figure 5, the effectiveness and complementarity of the three core mechanisms of Flat-IIN can be observed. Without any of these mechanisms, the SR of Flat-IIN is merely 0.560. As different mechanisms are progressively incorporated, both SR and SPL of Flat-IIN increase steadily. The introduction of the GB mechanism yields the most pronounced improvement in SR, indicating that GB effectively compensates for the agent’s false negative errors, i.e., missed detections during environment exploration. The incorporation of the VLM simultaneously improves both SPL and SR, demonstrating that the VFE mechanism effectively enhances the agent’s exploration efficiency, while the VLM score substantially aids the agent in object detection. The MASE mechanism enables the agent to observe instances from multiple viewpoints without backtracking, thereby determining whether an object of the same class as the target is indeed the target region. Consequently, although introducing the MASE mechanism leads to a lower SR than introducing the GB mechanism (0.649 vs. 0.689), the SPL achieved with MASE is higher than that with GB (0.274 vs. 0.269).

**Figure 6:**
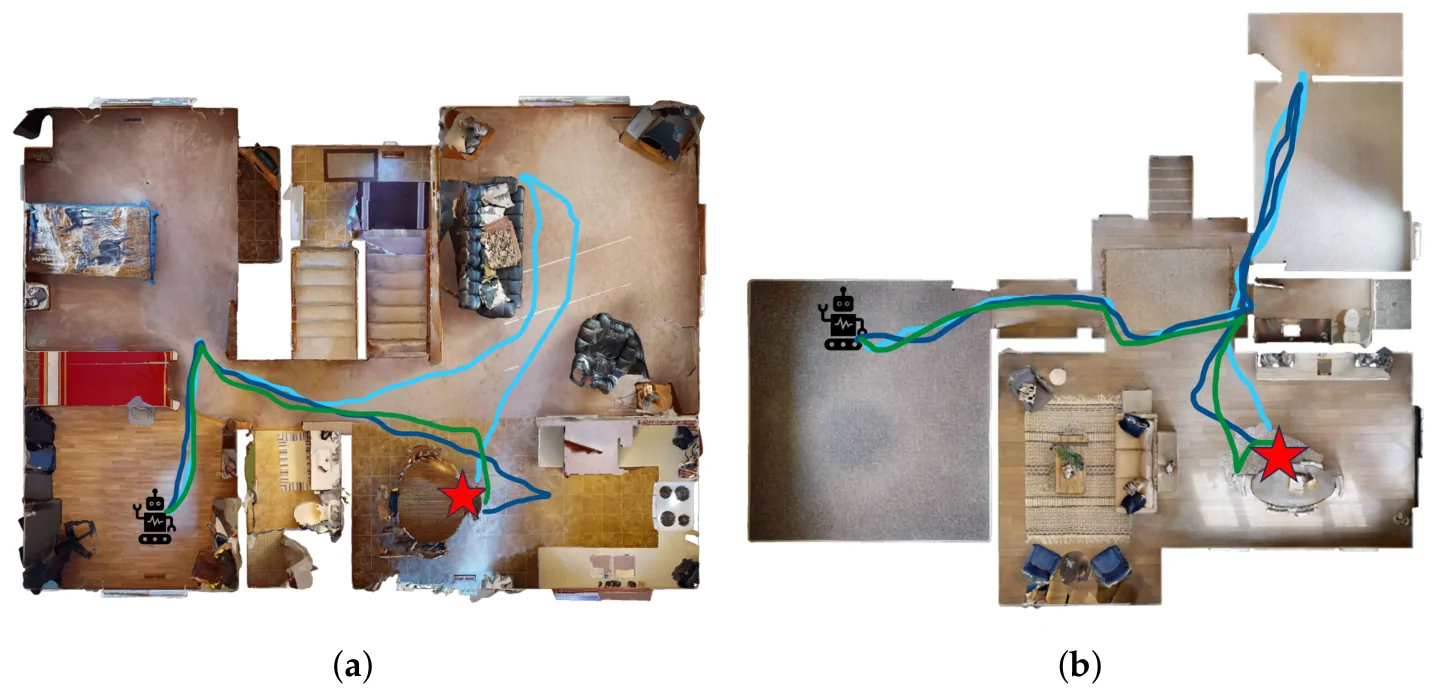
(**a**) Navigation trajectories of different methods in Scene a; (**b**) navigation trajectories of different methods in Scene b. Light blue denotes the navigation trajectory of Mod-IIN, dark blue denotes the navigation trajectory of Flat-IIN without VLM, and green denotes the navigation trajectory of Flat-IIN with VLM. Five-pointed stars indicate the target points.

To compare the difference between VLM-driven frontier exploration and classical frontier exploration, we select two episodes as examples and visualize the navigation trajectories of different methods, as shown in Figure 6. From Figure 6, it can be observed that VLM-driven frontier exploration navigates to the target more efficiently. This is because the VLM is able to recognize the target region from a distance and assign a VLM score, thereby guiding the agent to explore the environment.

### 5.7. Navigation Performance on Different Object Categories

**Figure 7:**
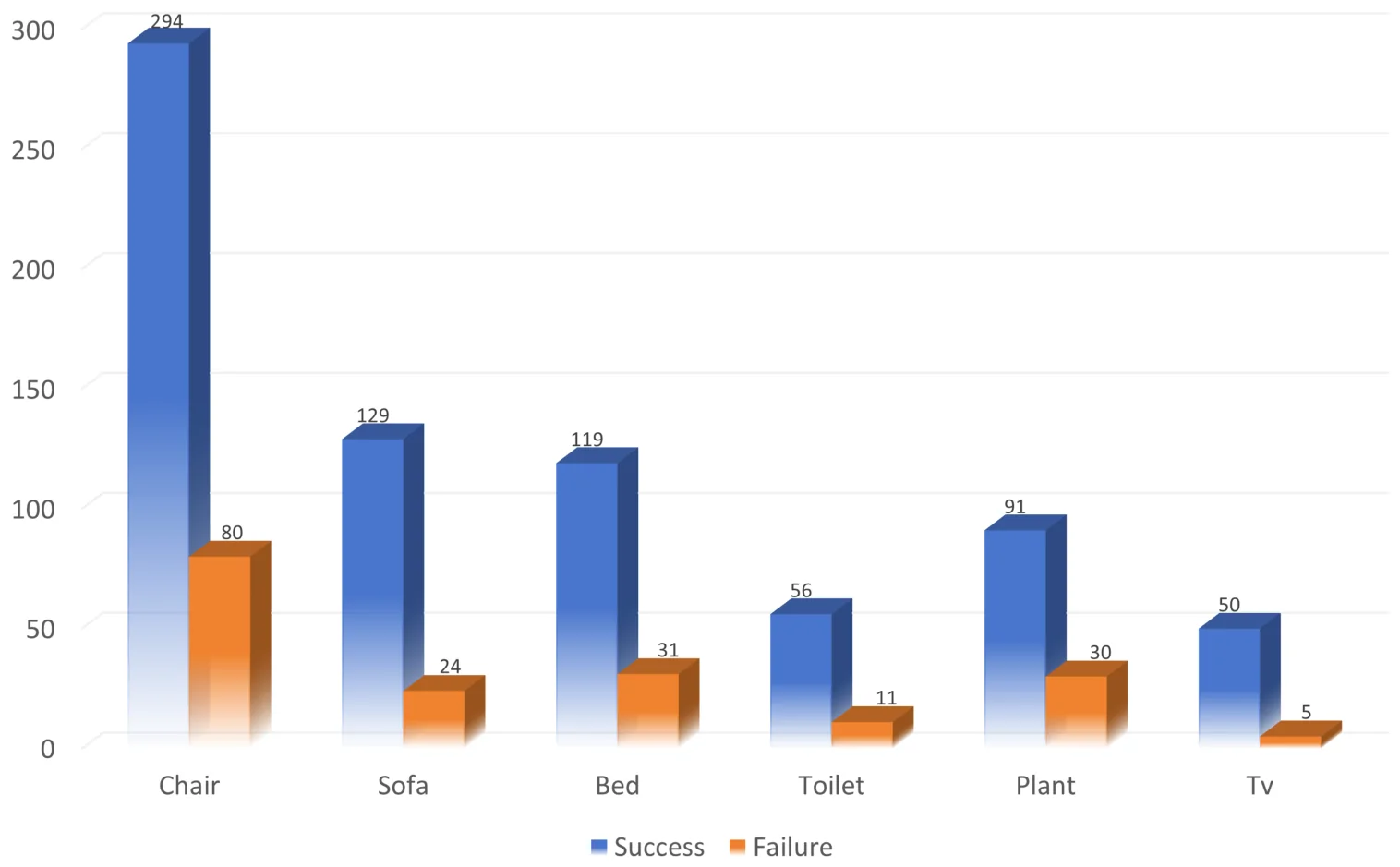
Number of successful and failed navigation episodes for each object category.

To evaluate the navigation performance of Flat-IIN across different object categories, we first count the episodes in which Detic successfully detects the target object, and record the numbers of successful and failed navigation episodes for each object category, as shown in Figure 7. The confidence threshold of Detic is set to 0.2. It can be observed from Figure 7 that the lowest SR occurs for the object category “plant” (75.2%). We attribute this to the fact that plants are typically small and lack distinctive visual features, making it difficult for the agent to effectively locate them during exploration. Moreover, the semantic relevance scores computed between the observations and the target object are generally low, leading to poor recognition. The relatively low SR for “chair” (78.6%) is mainly because multiple similar chairs often appear within the same scene, which can cause false positive failures, and because the agent may fail to precisely locate the region containing the target object. The SR for “bed” is 79.3%, with the primary failure reason being the agent’s inability to accurately localize the target object region. “Sofa,” as a target object, possesses distinct visual features, resulting in a relatively high SR (84.3%). Since “toilet” is usually located in bathroom areas, the agent can easily recognize the target object when exploring such regions; thus, its SR (83.5%) is higher than the average SR (79.4%). “TV,” as a target object, has prominent visual features and fewer distractor objects within the same scene, leading to a high SR of 90.9%. For episodes in which Detic failed to detect the target object category, the numbers of successful and failed episodes are 55 and 25, respectively, yielding an SR of 68.8%, which is lower than the average navigation SR, indicating that Detic detection failure leads to performance degradation.

### 5.8. Failure Case Analysis

**Figure 8:**
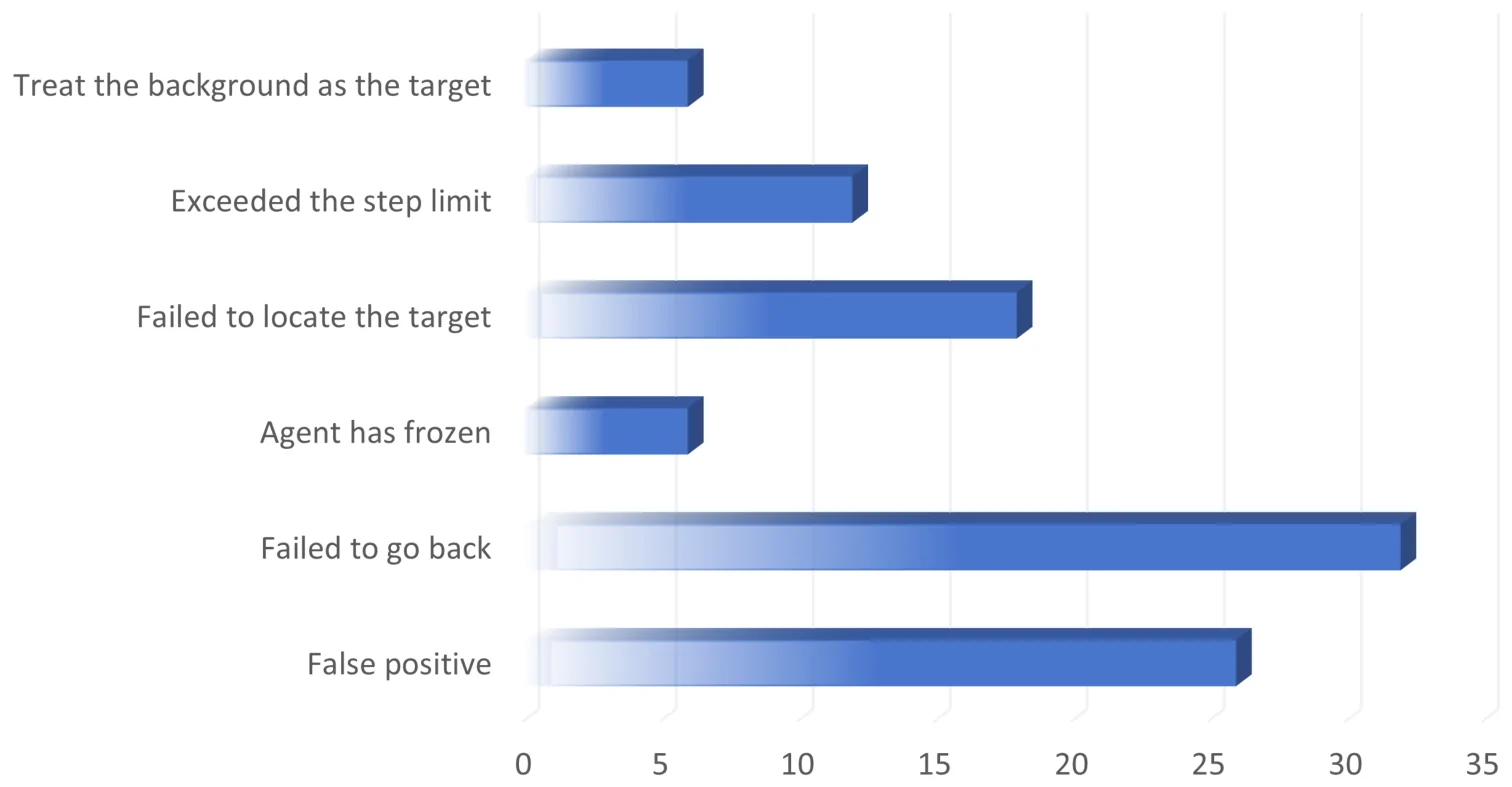
Comparison of failure case analysis.

We randomly sample 100 episodes from the failure cases of Flat-IIN and analyze their failure causes, as illustrated in Figure 8. As observed from the figure, the main causes of failure can be categorized into the following six types:

- False positive (26 occurrences): This refers to incorrectly identifying another object instance as the target object, which is primarily caused by erroneous detections produced by the dual-condition object detection mechanism.
- Failed to backtrack (32 occurrences): The main cause of backtracking failure is that the $M_{\mathrm{goal}}$ region with the highest semantic score $S_{\mathrm{sem}}$ after backtracking is not the actual navigation target.
- Agent freezing (6 occurrences): Errors in local path planning cause the agent to become stuck in a region and unable to move.
- Failed to locate the target (18 occurrences): The agent successfully detects the navigation target but fails to precisely localize it, resulting in a final distance exceeding 1 m from the target and thus failure.
- Exceeded the step limit (12 occurrences): The agent exceeds the 1000-step limit during navigation, leading to failure.
- Mistaking the background for the target (6 occurrences): The agent erroneously treats a background region of the target image as the navigation target, resulting in failure.

It is worth noting that “Failed to backtrack” is the largest failure category, but this phenomenon does not imply that the GB mechanism itself is the source of errors. The benefit of GB arises from the fact that, when the agent computes that no physically reachable exploration frontier exists on the local map, it no longer passively waits until the step budget is exhausted, but instead actively triggers the revisiting of candidate instances. This transforms episodes that would otherwise fail due to step-budget exhaustion into revisiting episodes with a chance of success. After revisiting, some episodes successfully recover the target, while others still fail because of the final $S_{\mathrm{sem}}$-based ranking. Therefore, GB is both the largest source of SR improvement and the largest failure category, and the two are not contradictory.

### 5.9. Real-World Experiments

**Figure 9:**
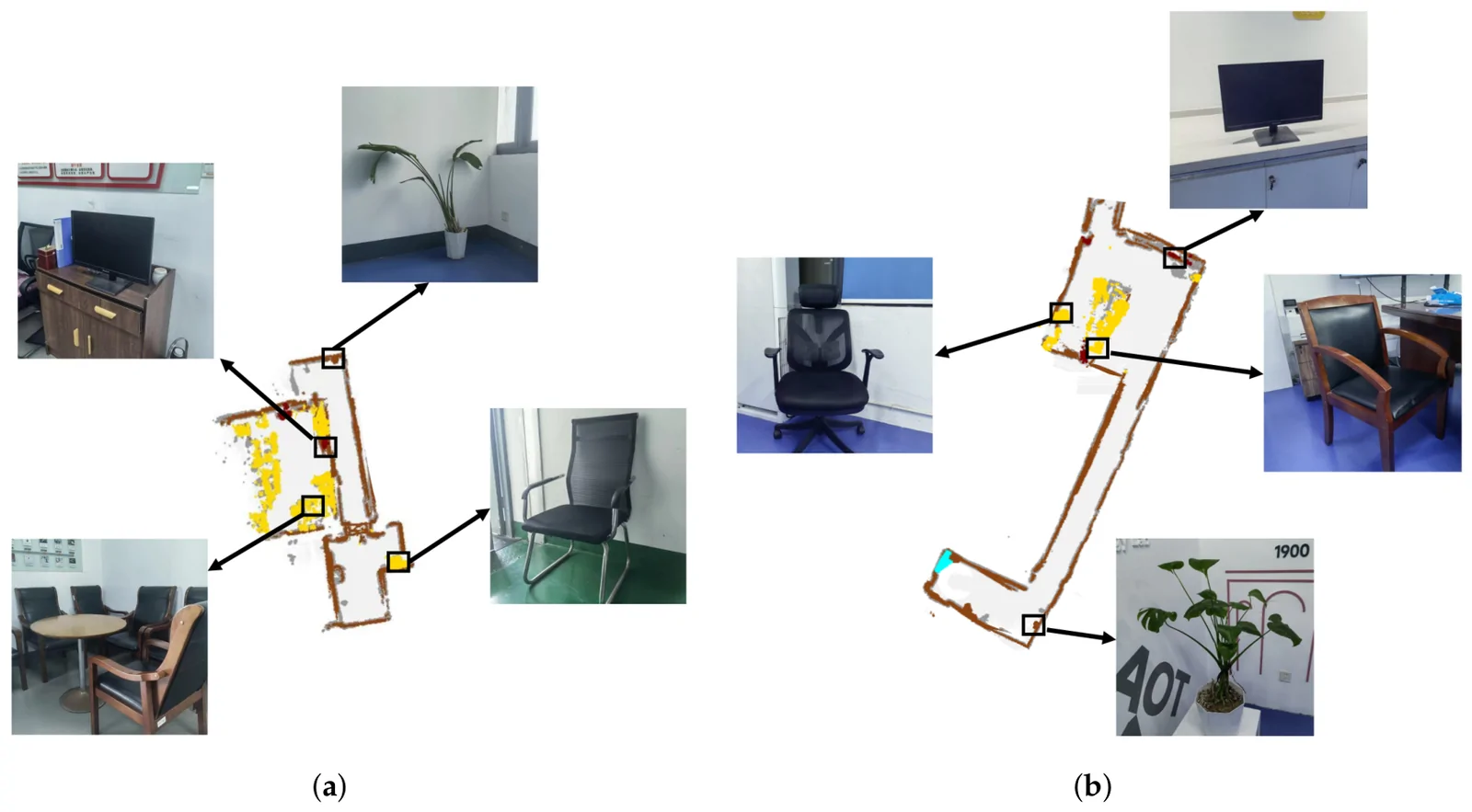
IIN in real-world scenarios. Scene (**a**) has an area of 86 m^2^, and Scene (**b**) has an area of 110 m^2^.

To evaluate the navigation performance of Flat-IIN in real-world scenarios, we select two environments as illustrated in Figure 9. In each environment, four target instances are chosen, and for each instance, five images captured from different viewing angles are used as the navigation target, resulting in 20 test trials per environment. The average SR and SPL are recorded, as shown in Table 8. From Table 8, it can be observed that in Scene A, Flat-IIN achieves an SR of 0.85 and an SPL of 0.39, both of which outperform Mod-IIN (0.80 SR and 0.33 SPL). In the larger Scene B, Flat-IIN achieves a 20% higher SR than the baseline. These results provide preliminary proof-of-concept evidence for the feasibility of Flat-IIN in real-world navigation. Meanwhile, tests show that Mod-IIN achieves a step execution rate of 0.71 steps/s, while Flat-IIN achieves 0.53 steps/s. The lower rate of Flat-IIN is mainly due to the additional VLM calls. In addition, during real-world deployment, the agent relies on wheel odometry for pose estimation. As the travel distance increases, cumulative localization drift is inevitable, causing misalignment between the 2D semantic map and the real environment, which in turn degrades the effectiveness of path planning and may even lead to navigation failure due to the absence of traversable paths. This result indicates that localization accuracy is one of the bottlenecks limiting navigation performance on real robotic platforms.

## 6. Conclusions

In this paper, we address the challenges of weak semantic perception, low exploration efficiency, and susceptibility to interference in target discrimination for the IIN task, and propose the Flat-IIN framework. Flat-IIN adopts 2D multi-channel semantic maps as a unified representation and incorporates three complementary core mechanisms: (1) MASE, which generates surround observation points via wavefront expansion and farthest point sampling, enabling the agent to verify candidate targets from multiple viewpoints; (2) GB, which leverages the co-occurrence distribution of objects in the target image as a spatial prior to perform secondary verification on missed candidate instances in the global map; and (3) VFE, which harnesses the powerful semantic understanding and commonsense reasoning capabilities of VLM to guide the agent to prioritize exploration of potential target regions. Experimental results demonstrate that Flat-IIN achieves an SR of 0.794, comparable to that of the best 3DGS method that relies on a pre-built map, while achieving the highest SR and SPL among the evaluated methods that do not rely on pre-built scene representations.

Future work will proceed along the following three directions: (1) improving real-world localization accuracy and maintaining semantic map consistency by integrating multi-sensor fusion with loop closure detection, pose graph optimization, and map registration; (2) reducing module dependencies to suppress error accumulation and enhance navigation robustness; and (3) exploring a system framework aimed at improving real-time navigation performance.

## Figures and Tables

**Table 1:** Experimental parameter settings of Flat-IIN.

Parameter	Value
α	1
β	0.5
γ	7
$\tau_{1}$	24.5
$\tau_{2}$	28.0
$w_{\mathrm{sem}}$	0.85
$w_{\mathrm{dist}}$	0.15

**Table 2:** Effect of different model combinations on Flat-IIN.

Instance Segmentation	VLM	SR	SPL
Mask R-CNN	Qwen-VL-Plus	0.788	0.331
	GLM-4.6V	0.794	0.338
YOLOv7 + MobileSAM	Qwen-VL-Plus	0.782	0.329
	GLM-4.6V	0.806	0.346

**Table 3:** Navigation data under different VLM invocation frequencies.

Frame Interval	SR	SPL	FPS	Token
1	0.770	0.342	0.73	253621
2	0.782	0.345	1.14	115873
3	0.798	0.351	1.28	81357
4	0.772	0.341	1.32	62792
5	0.764	0.337	1.47	49227
6	0.746	0.320	1.56	37628

**Table 4:** VLM score perturbation results.

Perturbation Probability *p*	SR	SPL
0.0	0.776	0.322
0.1	0.770	0.316
0.2	0.762	0.309
0.3	0.756	0.293
0.4	0.750	0.284
0.5	0.746	0.282

**Table 5:** Object detection under different conditions.

Condition	SR	SPL
Single	0.798	0.312
Dual	0.814	0.343

**Table 6:** Comparison with state-of-the-art methods on the IIN task.

Category	Method	SR	SPL
End-to-end	RL Baseline	0.083	0.035
	OVRL-v2-Image	0.006	0.002
	OVRL-v2-IIN	0.248	0.118
Modular	Mod-IIN	0.561	0.233
	UniGoal	0.602	0.237
	IEVE	0.684	0.241
3DGS	GaussNav	0.725	0.578
	GauScoreMap	0.784	0.605
Modular	Flat-IIN	0.794	0.331

**Table 7:** Ablation study results.

MASE	VFE	GB	SR	SPL
×	×	×	0.560	0.222
×	✓	×	0.591	0.254
✓	×	×	0.649	0.274
×	×	✓	0.689	0.269
✓	✓	×	0.691	0.293
×	✓	✓	0.706	0.286
✓	×	✓	0.751	0.295
✓	✓	✓	0.794	0.331

✓ indicates that the module is used; × indicates that the module is not used.

**Table 8:** Navigation results in real-world scenarios.

Scene	Method	SR	SPL
A	Mod-IIN	0.80	0.33
	Flat-IIN	0.85	0.39
B	Mod-IIN	0.55	0.20
	Flat-IIN	0.75	0.28

## Data Availability

Data is contained in this paper. The simulation experiments in this study are based on the publicly available Habitat-Matterport 3D (HM3D) dataset, which can be accessed at https://aihabitat.org/datasets/hm3d/ (accessed on 28 September 2026).
